# Dual Aurora A and JAK2 kinase blockade effectively suppresses malignant transformation

**DOI:** 10.18632/oncotarget.1615

**Published:** 2014-03-22

**Authors:** Hua Yang, Harshani R. Lawrence, Aslamuzzaman Kazi, Harsukh Gevariya, Ronil Patel, Yunting Luo, Uwe Rix, Ernst Schonbrunn, Nicholas J. Lawrence, Said M. Sebti

**Affiliations:** Drug Discovery Department, Chemical Biology and Molecular Medicine Program, Chemical Biology Core Moffitt Cancer Center and Research Institute; Departments of Molecular Medicine and Oncologic Sciences, University of South Florida, Tampa, FL, USA

## Abstract

Aurora A and JAK2 kinases are involved in cell division and tumor cell survival, respectively. Here we demonstrate that ectopic expression of Aurora A and JAK2 together is more effective than each alone at inducing non-transformed cells to grow in an anchorage-independent manner and to invade. Furthermore, siRNA silencing or pharmacological inhibition of Aurora A and JAK2 with Alisertib and Ruxolitinib, respectively, is more effective than blocking each kinase alone at suppressing anchorage-dependent and –independent growth and invasion as well as at inducing apoptosis. Importantly, we have developed dual Aurora and JAK inhibitors, AJI-214 and AJI-100, which potently inhibit Aurora A, Aurora B and JAK2 in vitro. In human cancer cells, these dual inhibitors block the auto-phosphorylation of Aurora A (Thr-288) and the phosphorylation of the Aurora B substrate histone H3 (Ser-10) and the JAK2 substrate STAT3 (Tyr-705). Furthermore, AJI-214 and AJI-100 inhibit anchorage dependent and independent cell growth and invasion and induce G2/M cell cycle accumulation and apoptosis. Finally, AJI-100 caused regression of human tumor xenografts in mice. Taken together, our genetic and pharmacological studies indicate that targeting Aurora A and JAK2 together is a more effective approach than each kinase alone at inhibiting malignant transformation and warrant further advanced pre clinical investigations of dual Aurora A/JAK2 inhibitors as potential anti tumor agents.

## INTRODUCTION

The Aurora family members of serine/threonine kinases, Aurora A, B and C, play key roles in the regulation of cell division. Aurora A regulates chromosome maturation and mitotic spindle formation, Aurora B controls chromosomal segregation and cytokinesis [1, 2] whereas Aurora C is involved in meiosis [3]. Aurora A, B and C kinases are found overexpressed in solid tumors, including colorectal, breast, and ovarian as well as leukemia [4, 5]. Over-expression of Aurora kinases is reported to be associated with genetic instability and tumor formation [6] and several lines of evidence implicate Aurora kinases in malignant transformation [7–9]. Therefore, these kinases are highly sought after as targets for the discovery of new anticancer drugs and intense efforts have been made to prepare specific pharmacological inhibitors [10]. For example, ZM44743911 [11] and Hesperadin [12] are Aurora kinase inhibitors that are more specific for Aurora B over Aurora A [12, 13]. VX-680, also known as MK-0457, was identified as a potent pan-Aurora inhibitor with Ki values of 0.6, 18, and 4.6 nM for Aurora A, C and B, respectively [14]. Although VX-680 has shown significant potential as an anti-cancer agent pre clinically, it failed in clinical trials due to cardiovascular side effects [15]. Other novel Aurora inhibitors that are undergoing clinical trials include MLN8054 and MLN8237 (Alisertib) [16], AZD1152 [17] and AT9283 (www.clinicaltrials.gov). Inhibition of Aurora kinase primarily leads to cell cycle arrest in the G2/M phase, but does not necessarily induce cell death. Therefore, it remains unclear whether Aurora kinase inhibitors will be effective as single agent or whether they will need to be combined with other agents. Several studies have reported on the benefits of combining Aurora kinase inhibitors with other anti-cancer agents such as cisplatin [18], temozolomide [19], taxanes [20], vorinostat [21] and nilotinib [22]. One notable example is the Aurora A inhibitor MLN8237, which overcomes resistance to BCR-ABL kinase inhibitors, in chronic myeloid leukemia (CML) [22].

The Janus kinases (JAK) family members, JAK1, JAK2, JAK3, and TYK2, are cytoplasmic protein tyrosine kinases that are required for signaling by receptors that lack intrinsic kinase activity such as the receptor for the cytokine interleukin 6 (IL-6) [23]. Some of the major substrates for the JAK family of kinases are the Signal Transducers and Activators of Transcription (STAT) proteins [24, 25]. JAK/STAT pathways are critically involved in the survival and proliferation of many cancer types [24, 26]. Furthermore, in some leukemias and myeloproliferative neoplasms, constitutive JAK2 activation (V617F mutation) drives malignant transformation [27] and this prompted a significant effort in targeting JAK2 inhibition as a potential therapeutic strategy. Ruxolitinib, a potent and selective JAK1/JAK2 inhibitor significantly inhibited interleukin-6 signaling and proliferation of cells that harbor JAK2-V617F mutation [28], and presently, is being investigated in clinic in patients with myeloproliferative neoplasms (MPNs) [29].

While Aurora kinases are involved in cell division and JAK2 kinase in tumor survival, whether these two kinases cooperate to induce malignant transformation is not known. Furthermore, it is not known whether human cancer cells require Aurora A and JAK2 alone or together to survive, to grow in an anchorage-dependent and –independent manner as well as invade and metastasize. In this manuscript, we demonstrate that Aurora A and JAK2 together are more effective than each alone at inducing non-transformed cells to grow in an anchorage-independent manner and invade. We also show that genetic depletion or pharmacological inhibition of Aurora A and JAK2 together is much more effective at inhibiting anchorage-dependent and –independent growth and invasion as well as at inducing apoptosis. Finally, we developed dual Aurora and JAK2 inhibitors that demonstrated potent inhibition of Aurora A, Aurora B and JAK2 in intact human cancer cells, induction of G2/M cell cycle accumulation and apoptosis as well as inhibition of anchorage-dependent and –independent proliferation, invasion and tumor growth in vivo.

## RESULTS

### Expression of Aurora A and JAK2 together is more effective than each alone at inducing non-transformed cells to grow in an anchorage-independent manner and invade

**Figure 1:**
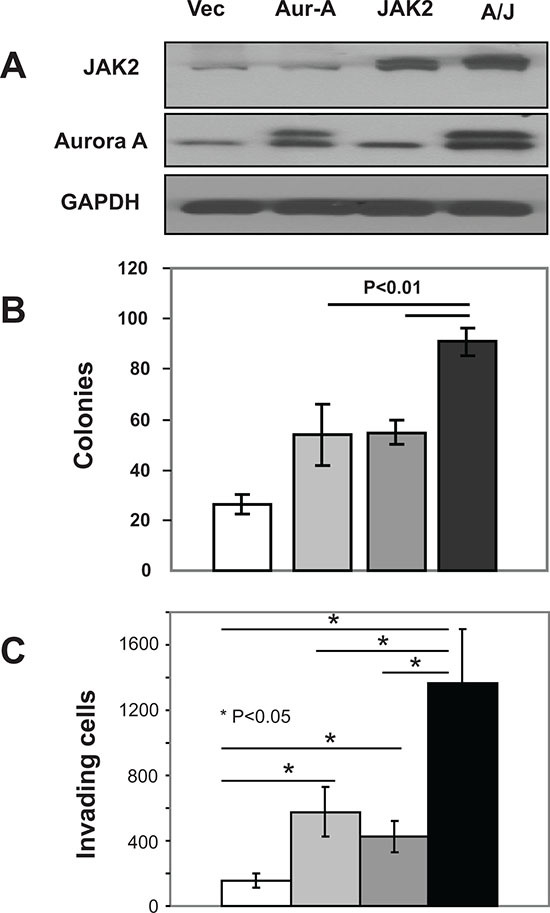
Co-expression of Aurora-A and JAK2 is more effective than single expression at inducing malignant transformation in HPNE cells HPNE cells were transfected with vector, Aurora A, JAK2 or both Aurora A and JAK2 as described in Materials and Methods. Cells were harvested 48 h post-transfection, and the resulting lysates were immunoblotted with indicated antibodies **(A)**, processed for soft agar growth assays **(B)** or invasion assays **(C)** as described in Materials and Methods. White, light gray, dark gray and black colors represent vector, Aurora A (Aur), JAK2 and Aurora A + JAK2 (A+J), respectively. For the soft agar **(B)**, the data are the average +/− SE of three wells per condition. For the invasion (C), the data are the average +/− SE of three independent experiments. The data for (A) and (B) are representative of three independent experiments, respectively.

While the potent antitumor activity of multi kinase inhibitors such as AT9283 that inhibit Aurora A and JAK2 among other kinases suggested that dual targeting of these two kinases is of benefit, direct evidence for this is lacking. Furthermore, whether these two kinases cooperate to induce malignant transformation is not known. To address this important question, we first transfected the non-transformed immortalized human pancreatic normal epithelial (HPNE) cells with either empty vector, Aurora A, JAK2 or Aurora A + JAK2 and determined the ability of these cells to grow in an anchorage-independent manner in soft agar and to invade through matrigel as described under Materials and Methods. Figure 1A shows that transfection of HPNE cells with Aurora A, JAK2 or Aurora A + JAK2 resulted in an increase in the expression of the corresponding proteins. Figure 1B shows that 26.3 colonies grew from empty vector transfected HPNE cells. In contrast, HPNE cells transfected with Aurora A, JAK2 and Aurora + JAK2 grew 54, 55, and 91 colonies, respectively. Similarly, Figure 1C shows that the number of HPNE cells that invaded following transfections with empty vector, Aurora A, JAK2 and Aurora A + JAK2 were 234, 869, 563 and 1665, respectively. These results demonstrate that Aurora A and JAK2 together are more effective than each is alone at promoting anchorage-independent growth and invasion in HPNE cells.

### Depletion of both Aurora A and JAK2 is highly effective at inhibiting anchorage-dependent and -independent growth and invasion and at inducing apoptosis

The results from Figure 1 suggested that Aurora A and JAK2 may cooperate to induce malignant transformation. To further support this suggestion, we reasoned that combined suppression of these two kinases may be more effective at inhibiting malignant transformation. To this end, we first used siRNA to deplete Aurora A, JAK2 or Aurora A + JAK2 in 8 human cancer cell lines from different lineages; three from breast (MDA-MB-468, MDA-MB-231 and MCF7), two from lung (A549 and H460), two from colon (HCT116 and HT29) and one from prostate (DU145); and determined the effects of these depletions on anchorage-dependent cell proliferation by MTT assays as described under Materials and Methods. Supplemental Figure S1 shows that depletion of either kinase alone had little effect on anchorage-dependent growth indicating that none of the cell lines depends on Aurora A or JAK2 alone for proliferation. In 5 out of the 8 cell lines (MCF-7, HCT-116, MDA-MB-231, H460 and A-549), even co-depletion was not highly effective at inhibiting anchorage-dependent growth. However, depleting both Aurora A and JAK2 together was more effective than depleting each alone in the remaining 3 cell lines (MDA-MB-468, HT-29 and DU-145) (Figure S1).

**Figure 2:**
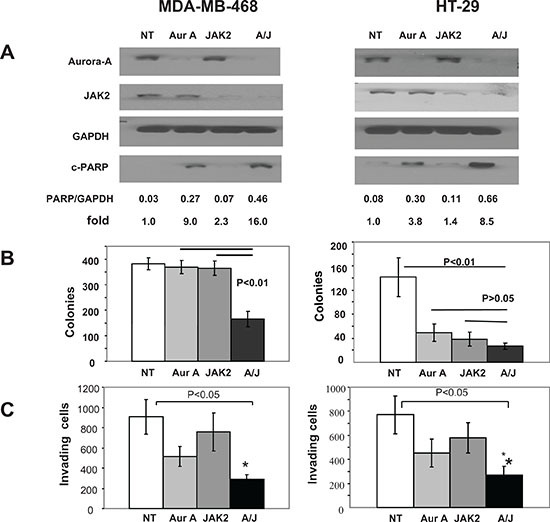
Depletion of Aurora A and JAK2 kinases is highly effective at inducing apoptosis and at inhibiting anchorageindependent growth and invasion MDA-MB-468 and HT-29 cells were transfected with NT siRNA, Aurora A siRNA, JAK2 siRNA or siRNAs to both aurora A and JAK2, and processed for Western blotting **(A)**, soft agar **(B)** or invasion **(C)** assays as described under Materials and Methods. For the soft agar **(B)**, the data are the average +/− SE of three wells per condition. For the invasion (C), the data are the average +/− SE of three independent experiments. The data for (A) and (B) are representative of three independent experiments, respectively.

We next focused on two of these cell lines, MDA-MB-468 and HT-29, to determine the requirements of Aurora A and JAK2 on survival, anchorage-independent growth and invasion. To this end, we silenced Aurora A, JAK2 or Aurora A + JAK2, and processed the cells for Western immunoblotting, soft agar and invasion assays as described in Materials and Methods. Figure 2A shows that in MDA-MB-468, depletion of Aurora A, JAK2 and Aurora A + JAK2 induced PARP cleavage relative to NT by 9.0, 2.3 and 16.0 fold, respectively. Similarly, in HT-29 cells, depletion of Aurora A, JAK2 and Aurora A + JAK2 induced PARP cleavage relative to NT by 3.8, 1.4 and 8.5 fold, respectively. Thus, depletion of both kinases is more effective than depleting each alone at inducing apoptosis as measured by PARP cleavage.

Figure 2B shows that MDA-MB-468 cells transfected with NT, siRNA to Aurora A, JAK2 and Aurora A + JAK2 grew 382, 369, 365 and 165 soft agar colonies, respectively. Thus, depleting MDA-MB-468 cells from Aurora A, JAK2 or both resulted in 3.5%, 4.5% and 56.9% inhibition of soft agar growth, respectively. Furthermore, Figure 2B also shows that HT-29 cells transfected with NT, siRNA to Aurora A, JAK2 and Aurora A + JAK2 grew 142, 49, 39 and 27 soft agar colonies, respectively. Thus, depleting HT-29 cells from Aurora A, JAK2 or both resulted in 65.5%, 72.6% and 81% inhibition of soft agar growth. Therefore, the data suggest that the combination knockdown of Aurora A and Jak2 kinases has divergent effects on anchorage-independent growth. MDA-MB-468 cells do not appear to require each kinases alone but do require both kinases for growth on soft agar. In contrast, HT29 cells appear to require Aurora A and JAK2 individually for anchorage-independent growth.

We next determined the effect of knockdown of Aurora A and JAK2 on invasion of MDA-MB-468 and HT29 cells. Figure 2C shows that in MDA-MB-468 cells, 1031 cells invaded in the NT-transfected sample. Transfection with JAK2 siRNA had very little effect on invasion (994 invaded cells) whereas transfection with Aurora A siRNA inhibited invasion by 41.3% (605 invaded cells). Depletion of both kinases was more effective and inhibited invasion by 63.6 % (375 invaded cells). Similar results were obtained with HT29 cells where the number of invading cells were 1815, 1994, 1140 and 717 in NT, JAK2, Aurora A and Aurora + JAK2 transfected cells, respectively. Thus, in both cell lines, knocking down JAK2 had no effect but depleting both kinases was more effective than each was alone.

### Combination treatment with the Aurora A inhibitor Alisertib and the JAK2 inhibitor Ruxolitinib is more effective than single treatment at inhibiting anchorage-dependent and -independent proliferation and invasion and at inducing apoptosis

Figure 2 demonstrated that depleting Aurora A and JAK2 together is more effective than depleting each alone at suppressing malignant transformation. We next used a pharmacological approach to further investigate the effects of inhibiting these 2 kinases alone and together on malignant transformation. To this end, we first treated MDA-MB-468 and HT29 cells with various concentrations of the Aurora A kinase inhibitor Alisertib and the JAK2 inhibitor Ruxolitinib and determined their IC50 values for anchorage-dependent growth as described under Methods. Supplemental Figure S2A shows that Alisertib and Ruxolitinib inhibited anchorage-dependent proliferation in a concentration dependent manner with IC 50 values for Alisertib of 0.07 and 2.0 μM and for Ruxolitinib of 26.0 and 44.7 μM in MDA-MB-468 and HT29, respectively. Figure S2B shows that treatment of MDA-MB-468 cells with vehicle, Alisertib (0.05 μM), Ruxolitinib (35 μM) or the combination inhibited proliferation by 25.4%, 51.2% and 80.1%, respectively. Treatment of HT29 cells with vehicle, Alisertib (0.05 μM), Ruxolitinib (20 μM) or the combination inhibited proliferation by 5%, 14.3% and 45.4%, respectively. Thus, combination of Alisertib and Ruxolitinib was more effective than single treatment at inhibiting anchorage-dependent proliferation.

**Figure 3:**
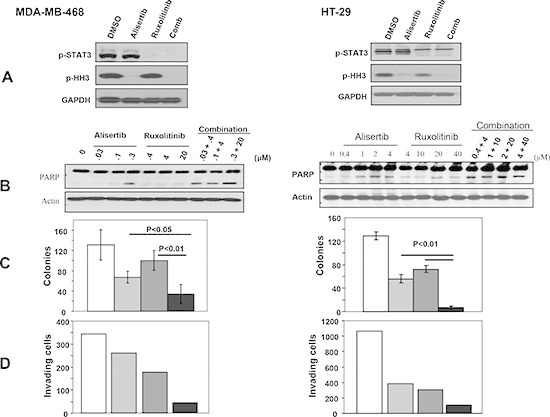
Combination treatment with the Aurora A inhibitor, Alisertib, and the JAK2 inhibitor, Ruxolitinib, is more effective than single treatment at inhibiting anchorage-independent growth and invasion and at inducing apoptosis MDA-MB-468 and HT-29 cells were treated with vehicle control (white), Alisertib (light gray), Ruxolitinib (dark gray) or the combination (black), and the Cells were processed for Western blotting **(A)** and **(B)**, soft agar **(C)** and invasion **(D)** assays as described under Materials and Methods. For the soft agar (C), the data are the average +/− SE of three wells per condition. For the invasion (D), the data are from one invasion chamber per condition. The data for (A), (B), (C) and (D) are representative of three, two, three and two independent experiments, respectively.

To determine the effects of these inhibitors on apoptosis induction, we treated MDA-MB-468 and HT29 cells with various concentrations of Alisertib, Ruxolitinib either alone or in combination and processed the cells for Western blotting as described under Materials and Methods. Figure 3A shows that treatment of MDA-MB-468 and HT-29 cells with Alisertib (0.5 and 2 μM, respectively) inhibited P-Histone H3 but not P-STAT3, whereas treatment with Ruxolitinib (30 and 20 μM, respectively) inhibited P-STAT3 but not P-Histone H3. Figure 3B shows that in MDA-MB-468 cells, neither Alisertib nor Ruxolitinib induced PARP cleavage when used as single agents at low concentrations (for example, Alisertib (0.03 μM) and Ruxolitinib (0.4 μM). However, when these low concentrations were used in combination they induced PARP cleavage (Figure 3B). Similar results were obtained with HT-29 cells (Figure 3B). Thus, consistent with the anchorage-dependent proliferation results of Figure S2, pharmacological inhibition of Aurora A and JAK2 combined is more effective than single kinase inhibition at inducing apoptosis.

We next determined whether combination treatment with Alisertib and Ruxolitinib is more effective than single agent treatment toward inhibiting anchorage-independent growth of MDA-MB-468 and HT-29 cells in soft agar as described under Materials and Methods. Figure 3C shows MDA-MB-468 cells treated with vehicle, Alisertib (0.5 μM), Ruxolitinib (30 μM) or both compounds grew 131, 67, 100, and 34 colonies, respectively. Thus, the combination was more effective (74%) than Alisertib (49%) and Ruxolitinib (23.7%) at inhibiting anchorage-independent growth. Figure 3C also shows that HT-29 cells treated with vehicle, Alisertib (2 μM), Ruxolitinib (20 μM) or both compounds had 129, 56 (56.6% inhibition), 72 (44.2% inhibition), and 7 colonies (94.6% inhibition), respectively.

Next, we determined the effects of the combination treatment of Alisertib and Ruxolitinib on invasion of MDA-MB-468 and HT-29 cells, using Matrigel coated Transwell cell culture chambers as described under Materials and Methods. Figure 3D shows that MDA-MB-468 cells treated with vehicle, Alisertib (0.5 μM), Ruxolitinib (30 μM) or both compounds had 344, 260, 177 and 42 invaded cells, respectively. Thus, the combination was more effective (87.8%) than Alisertib (24.5 %) and Ruxolitinib (48.6%) at inhibiting invasion. In HT-29 cells, treatment with vehicle, Alisertib (4 μM), Ruxolitinib (40 μM) or both compounds resulted in 1066, 384 (64% inhibition), 305 (71.4% inhibition) and 110 (89.7% inhibition) invaded cells, indicating that pharmacological inhibition of Aurora A and JAK2 combined is more effective than single kinase inhibition at suppressing the invasive capacity of these cancer cells.

### Discovery of AJI-214 and AJI-100, highly potent small-molecule dual inhibitors of Aurora and JAK2 kinases

**Figure 4:**
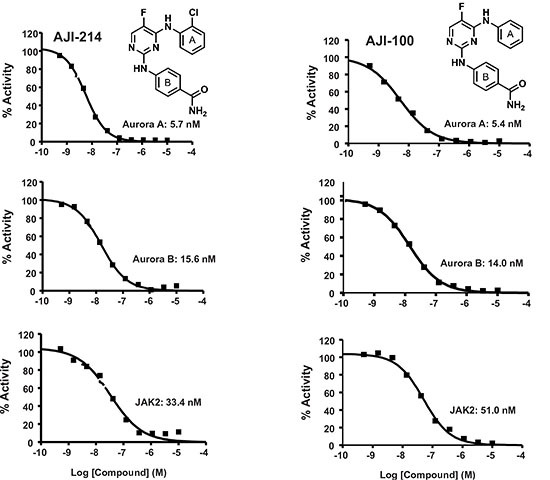
AJI-214 and AJI-100 are dual Aurora A/B and JAK2 inhibitors AJI-214 and AJI-100 synthesized as described by us previously [30] and submitted to Reaction Biology Inc. for determination of IC50 values against Aurora A, Aurora B and JAK2 using the HotSpot method as described previously [30].

**Figure 5:**
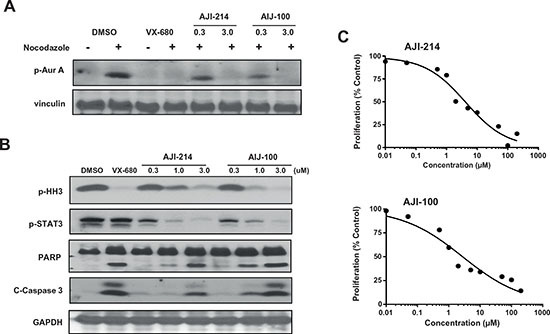
AJI-214 and AJI-100 inhibit the phosphorylation of Aurora A, Histone H3 and STAT3, induce apoptosis and inhibit anchorage-dependent proliferation in cancer cells **(A)** MDA-MB-468 Cells were synchronized by treatment with nocodazole (100 ng/mL) for 20 h, treated with AJI-214 or AJI-100 for 2 h and processed for Western immunoblotting with p-Aurora-A (Thr288) antibody as described under Materials and Methods. VX-680 was used as a control for Aurora-A inhibition. **(B)** MDA-MB-468 Cells were treated with AJI-214 or AJI-100 for 2 h and processed for Western immunoblotting with P-HH3, P-STAT3, PARP, C-Caspase3 and GAPDH antibodies as described under Materials and Methods. **(C)** MDA-MB-468 Cells were plated in 96-well plates and treated with the indicated concentrations of AJI-214 and AJI-100 for 48 h and processed for MTT assays as described in “Materials and Methods”. The data for (A), (B) and (C) are representative of two, two and three independent experiments, respectively.

Figure 1 provided evidence for the cooperation between Aurora A and JAK2 to induce malignant transformation, whereas Figures 2 and 3 demonstrated using 2 approaches, genetic knockdown and pharmacological inhibition, that blocking these 2 kinases together is more effective than blocking each alone at suppressing malignant transformation. These results prompted us to develop dual Aurora A and JAK2 inhibitors. To this end, recently our in-house chemistry and structural biology efforts have yielded highly potent chlorophenyl substituted pyrimidine-based Aurora kinase inhibitors [30]. Two analogues, AJI-214 and AJI-100 (Figure 4) were identified as dual inhibitors of Aurora A and JAK2 kinases. The two analogues differ only by a single substitution of a chlorine in AJI-214 by a hydrogen in the ortho position of the phenyl ring A of AJI-100 (Figure 4). AJI-214 and AJI-100 inhibited Aurora A in vitro (IC50 = 5.7 nM and 5.4 nM) and Aurora B (15.6 nM and 14.0 nM) (Figure 4). In addition, both inhibitors also inhibited potently JAK2 (33.4 nM and 51 nM) (Figure 4). To determine whether the two analogues inhibit their targets in intact cancer cells, we treated MDA-MB-468 cells with increasing concentrations of AJI-214, AJI-100, or control Aurora inhibitor VX-680, and determined their effects on Aurora A auto-phosphorylation (Aurora A is autophosphorylated on its activation loop on Thr-288 during mitosis [31]), on phosphorylation of Histone H3 on Ser-10 (Aurora B substrate) and on phosphorylation of STAT3 at Y705 (JAK2 substrate). For detection of Aurora A kinase activity in intact cells, the cells were first synchronized by pretreatment with nocodazole for 20 hr prior to treatment with inhibitors as described under Materials and Methods. Figure 5A shows that AJI-214 and AJI-100 suppressed nocodazole-induced Aurora A auto-phosphorylation on Thr-288 at concentrations between 0.3 and 3 μM. Furthermore, AJI-214 and AJI-100 decreased the phosphorylation of the Aurora B substrate Histone H3 and the phosphorylation of the JAK2 substrate STAT3 in a concentration-dependent manner (Figure 5B). The Aurora kinase inhibitors VX-680 and Alisertib were highly effective at inhibiting auto-phosphorylation of Aurora A and phosphorylation of Histone H3 but not the phosphorylation of STAT3 in MDA-MB-468 cells (Figure 5A and Supplementary Figure S3). In contrast, the JAK2 inhibitor Ruxolitinib was able to inhibit the phosphorylation of STAT3 but not that of Histone H3 or the auto-phosphorylation of Aurora A (Figure S3). Similar results were obtained in HEL leukemia cells that harbor a JAK2-V617F mutation and the resulting hyper-phosphorylated STAT3 on Tyr-705 (Figure S3C). Furthermore, AJI-214 is selective for Aurora and JAK2 over other kinases as demonstrated by the observation that it did not decrease the levels of P-Akt and P-Erk (Figure S3B). Taken together, these findings demonstrated that the novel dual inhibitors AJI-214 and AJI-100 potently inhibit the activities of Aurora A, Aurora B and JAK2 in intact human cancer cells.

### AJI-214 and AJI-100 inhibit anchorage-dependent and –independent cell growth and invasion as well as induce apoptosis in human cancer cells

**Figure 6:**
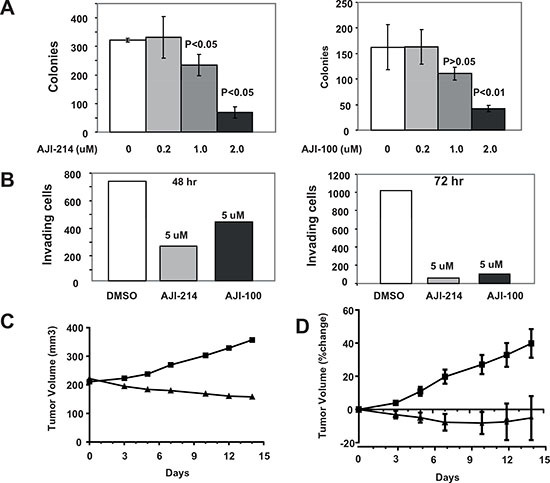
Aurora A and JAK2 dual inhibitors are highly effective at inhibiting anchorage-independent growth and invasion as well as in vivo tumor growth of MDA-MB-468 xenografts in nude mice MDA-MB-468 cells were treated with vehicle control, AJI-214 or AJI-100 and the cells were processed for soft agar **(A)** and invasion **(B)** assays as described under Materials and Methods. For the soft agar **(A)**, the data are the average +/− SE of three wells per condition. For the invasion (B), the data are from one invasion chamber per condition. The data for (A) and (B) are representative of three and two independent experiments. **(C** and **D) Effects of AJI-100 on tumor growth of MDA-MB-468 xenografts in nude mice.** Mice bearing MDA-MB-468 tumor xenografts were treated (once a day for 14 days) with vehicle (50% PG+15%HPCD) or AJI-100 (50 mpk/day) as described under Materials and Methods. (C) Representative tumor growth curve from vehicle treated mouse (square) and AJI-100 treated mouse (triangle). **(D)** Average (+/− SE) percent change in tumor volumes for each treatment group (vehicle squares, AJI-100 triangles).

Figures 4, 5A and 5B demonstrated that AJI-214 and AJI-100 are potent dual Aurora and JAK2 inhibitors. In addition, Figures 2 and 3 demonstrated the benefits of inhibiting both Aurora A and JAK2 at suppressing malignant transformation. Therefore, we next determined the effects of these dual inhibitors on anchorage-dependent and -independent tumor cell growth, apoptosis, invasion and cell cycle progression as described under Materials and Methods. Figure 5C shows that AJI-214 and AJI-100 inhibited MDA-MB-468 anchorage-dependent growth in a concentration dependent manner with IC50 values of 4.6 and 3.6 μM, respectively. Next, we evaluated whether the inhibitors can induce apoptosis by treating MDA-MB-468 cells with the inhibitors and processing the cells for Caspase 3 activation and PARP cleavage by Western blotting as described under Materials and Methods. Figure 5B shows that the two inhibitors induced Caspase 3 activation and PARP cleavage in a dose-dependent manner. The ability of AJI-214 and AJI-100 to inhibit anchorage–independent growth was evaluated in soft agar as described under Materials and Methods. Figure 6A shows that AJI-214 and AJI-100 inhibited colony formation of MDA-MB-468 cells in soft agar with increasing concentrations. The effects of AJI-214 and AJI-100 on invasion were also evaluated as described under Materials and Methods. Figure 6B shows that MDA-MB-468 cells treated with vehicle, AJI-214 (5 μM) and AJI-100 (5 μM) for 48 hr had 723, 252 (65.2% inhibition) and 426 (41.1% inhibition) invaded cells, respectively. Cells treated for a longer period (72 hr), had 1020, 56 (94.5% inhibition), and 104 (89.8% inhibition) invaded cells, respectively.

Since Aurora A and B are intimately involved in mitosis, we evaluated the effect of AJI-214 on cell-cycle progression in MDA-MB-468 cells by flow cytometry analysis as described under Materials and Methods. Supplemental Figure S4 shows that AJI-214 treatment at 3 μM for 24 hr induced a 2-fold increase in the proportion of MDA-MB-468 cells in the G2/M phase of the cell cycle. AJI-214 also induced G2/M accumulation (4-fold increase) in HeLa cells (data not show). Furthermore, the Aurora kinase inhibitor VX-680 but not the JAK2 inhibitor Ruxolitinib induced a significant G2/M accumulation in the same MDA-MB-468 cells under the same conditions (data not shown), suggesting that AJI-214 mediated-G2/M accumulation is mainly dependent on its ability to inhibit Aurora not JAK2.

### AJI-100 induces tumor regression in vivo

We next determined whether AJI-100 is able to inhibit tumor growth in vivo in the nude mouse xenograft model. AJI-214 was not soluble at the concentrations needed for in vivo studies. For these studies, MDA-MB-468 cells were implanted s.c. under the flank of nude mice and when the tumors reached an average volume of 250 mm^3^, the mice were randomized and injected i.p. daily for 14 days with either vehicle or AJI-100 (50 mpk). Figure 6C shows representative tumor growth curves. In the vehicle-treated mouse the tumor grew from 211 to 357 mm^3^ over the 14 day treatment period, whereas the tumor from the AJI-100-treated mouse regressed from 222 to 158 mm3 (Figure 6C). Figure 6D shows the average % change in tumor volumes from all the mice treated. On average tumors from the vehicle-treated mice grew by 40 % whereas those from the AJI-100-treated mice regressed by 9% (Figure 6D). The average % change in tumor volumes from vehicle and the AJI-100 treated groups were statistically different for measurements on days 3 (p= 0.04), 5 (p=0.003), 7 (p=0.002), 10 (p=0.004), 12 (p=0.018) and 14 (p=0.025). Treatment with either vehicle or AJI-100 had little effect on mouse weights, which increased by 3.33 % and 0.04 %, respectively, over the 14-day treatment period.

## DISCUSSION

Aurora kinases play important roles in the regulation of mitosis. Aurora A is required for spindle assembly, whereas Aurora B is required for histone H3 phosphorylation, chromosome segregation, and cytokinesis [1, 2]. Aurora kinase inhibitors are of considerable interest as potential anticancer agents because of significant anti-tumor activity reported in preclinical studies either as single agents or in combination with other anti-cancer drugs [7, 14, 15, 32–34]. Furthermore, the JAK/STAT pathway has also attracted interest as a therapeutic target for human solid tumors as well as hematological malignancies [24, 26, 35, 36]. Previous reports have shown that multi kinase inhibitors such as AT9283 and CEP-701 that block Aurora A and JAK2 among other kinases inhibit tumor growth potently (see more below). However, the extent to which the dual inhibition of these 2 kinases contributes to the anti tumor activity of these agents is not known. Furthermore, it is also not known whether some human tumors depend on both Aurora A and JAK2 for survival and malignant transformation. Finally, whether Aurora A and JAk2 cooperate to induce malignant transformation and whether dual targeting is more effective than single targeting are also not known. In this manuscript, we used genetic and pharmacological approaches and provided evidence for the superiority of dual Aurora A and JAK2 targeting. First, we demonstrated that Aurora A and JAK2 together are more effective than each alone at inducing non-transformed cells to grow in a non-adherent manner and invade. Since both anchorage-independent tumor growth and invasion facilitate metastasis, our results suggest that aberrantly-activated Aurora A and JAK2 pathways may cooperate to promote tumor metastasis. The observation that Aurora A increased anchorage-independent growth and invasion is consistent with previous reports [37]. Furthermore, the JAK2-induced soft agar growth and invasion may be mediated by activation of STAT3 that has also been implicated in metastasis [38, 39]. Our results demonstrating that the co-expression of Aurora A and JAK2 is more effective than single expression at inducing anchorage-independent growth and invasion prompted us to evaluate the effectiveness of the dual inhibition of these two kinases on malignant transformation. Both approaches we used, combination silencing by siRNA as well as combination treatment with Aurora A and JAK2 pharmacological inhibitors demonstrated the superior activity of dual suppression. Indeed, in 3 out of the 8 cell lines evaluated, depletion of both kinases was much more effective than single depletion at inhibiting anchorage-dependent growth. Furthermore, the finding that dual silencing of both kinases was more effective at inducing apoptosis and inhibiting anchorage-independent growth and invasion suggested that some cancer cells depend on both kinases for maintaining malignant transformation and that treatment targeting both Aurora A and JAK2 pathways might be a more effective anti cancer therapy approach. The clinical validation of this approach is feasible since the inhibitors of these two kinases, Alisertib and Ruxolitinib, that we have used to demonstrate the dual inhibition benefit, are presently undergoing clinical trials as single agents [40–42].

At present we do not know what makes some tumors (i.e. MDA-MB-468, HT-29 and DU-145) but not others (A549, MCF7, H460, MDA-MB-231 and HCT-1116) more susceptible to Aurora/JAK2 dual targeting. Differences in the expression levels of these two kinases cannot account for this since we have seen no correlations between the expression levels with sensitivity to dual targeting (data not shown). Studies comparing differences between a large number of susceptible and non-susceptible human cancer cell lines with respect to gene expression profiling, proteomic and phopho-proteomic are warranted for indentifying the underlying mechanisms.

While combination treatment with 2 or more selective agents is very useful for proof of concept studies in preclinical settings, it has drawbacks and presents serious challenges in human clinical trials. The most critical are off-target toxicities associated with each compound that necessitate separate early phase clinical trials to assess single agent safety. Furthermore, subsequent phase I trials with safe and tolerable doses of each compound may result in unacceptable toxicities of the combination. Thus, while a therapy based on a combination of distinct Aurora A and JAK2 inhibitors is feasible, there are obvious advantages to using a single drug that inhibits both kinases. In this manuscript, we report on the discovery of dual inhibitors that potently inhibit Aurora A and B as well as JAK2 in vitro and in intact human cancer cells, and that are highly effective at inhibiting anchorage-dependent and –independent growth and invasion and at inducing cell cycle arrest and apoptosis. Others have also reported recently on inhibitors that target several kinases including JAK2 and Aurora kinases. For example, AT9283 is a multi-targeted kinase inhibitor with in vitro IC50 values for JAK2, JAK3, Aurora A, Aurora B and Abl of 1.2, 1.0,1.0, 3.0, 3.0, and 4.0 nM, respectively [43]. AT9283 was shown to be effective at suppressing the growth in nude mice of BCR-ABL positive human CML cells as well as increasing survival of mice inoculated with cells from CML patients [44]. AT9283 is currently in Phase I human clinical trials [45]. Another example is that of the staurosporine analog CEP-701 that inhibits several kinases including FLT3 (IC50 = 3 nM), Aurora A (IC50 = 2.7 nM), Aurora B (IC50 = 6.9 nM) and JAK2 (IC50 = 1.2 nM) among others [46]. A recent report showed that in colony forming assays, JAK2- selective inhibitors inhibit the growth of erythroid colonies, and Aurora-selective inhibitors inhibit myeloid colony growth, whereas CEP-701 inhibited both [47]. Based on this observation, the authors suggested that CEP-701 may accomplish this through inhibition of Aurora and JAK2. CEP-701 is currently being investigated in clinical trials in patients with primary or post-polycythemia vera/essential thrombocythemia myelofibrosis [48].

The dual action of our inhibitors was further confirmed by demonstrating that the selective Aurora inhibitors VX-680 and Alisertib and the selective JAK2 inhibitor Ruxolitinib impaired only their respective targets pT288-Aurora A and pSer10-Histone H3 (Aurora A and B) and pY705-STAT3 (JAK2) whereas AJI-214 and AJI-100 inhibited both targets. Furthermore, AJI-214 and VX-680 but not Ruxolitinib induced G2/M accumulation, suggesting that this affect is due to the ability of AJI-214 to inhibit Aurora but not JAK2. The G2/M arrest seen with AJI-214 is consistent with previous studies showing that inhibition of Aurora kinases interferes with cell cycle progression primarily through a G2/M arrest [49].

Taken together, our multi-pronged approach using ectopic co-expression to demonstrate cooperation of Aurora A and JAK2 to transform normal cells as well as the genetic silencing and the pharmacological inhibition of these kinases in human cancer cells, support the notion that the combination treatment targeting both Aurora A and JAK2 pathways might be more beneficial to some cancer patients.

## MATERIALS AND METHODS

### Cell lines and reagents

Human breast cancer cell lines (MDA-MB-468, MDA-MB-231 and MCF-7), lung cancer cell lines (A549 and H460), and colon cancer cells (HCT-116 and HT-29) were obtained from ATCC (the American Type Culture Collection, Manassas, VA, USA). Cells were grown in Dulbecco's modified Eagle's medium (DMEM) or RPMI 1640, containing 10% heat-inactivated fetal bovine serum. Alisertib (MLN8237) and INCB-18424 (Ruxolitinib) were purchased from Selleck chemicals (Houston, TX). Nocodazole and thiazolyl blue tetrazolium bromide (MTT) were obtained from Sigma-Aldrich (St. Louis, MO). VX-680 (Tozasertib) was purchased from BioVision incorporated (Milpitas, CA). AJI-214 and AJI-100 were synthesized as described previously [30]. All drugs for cell culture were dissolved in DMSO (Sigma).

### Knockdown by siRNA transfection

Cancer cells were plated in six-well plates and next day transiently transfected with 35 nM of SignalSilence siRNA for Aurora A/AIK, JAK2 (Cell Signaling #8883 and #6235), or nonspecific control siRNA (Cell Signaling #6568) using the Lipofectamine RNAiMAX reagent (Invitrogen #13778), according to the manufacturer's instructions. Transfected cells were collected 72 h after transfection and protein expression levels were monitored by Western blot analysis.

### Ectopic expression of Aurora A and JAK2 in HPNE cells

HPNE cells were obtained from Drs Der and Campbell (University of North Carolina), and were originally isolated from the ductal structure of a normal human pancreas and were immortalized with the catalytic subunit of telomerase (h-Tert), as was described previously [50]. HPNE cells were maintained in Medium D that is comprised of one volume of M3 base (InCell Corp., San Antonio, TX, USA), three volumes of glucose-free DMEM, and 5% FBS, 5.5mM glucose, 10ng/ml EGF, and 50μg/ml gentamycin, at 37°C in humidified atmosphere containing 5% CO2. HA-wt-Aurora A was created by PCR using pcDNA3-Aurora A as template, as previously described [9] and Flag-tagged JAK2 was purchased from GeneCopoeia (Rockville, MD). Transfections were performed using 0.3 ugs of DNA constructs in Lipofectamine 2000 (Invitrogen).

### Cell viability and proliferation assays

Cells were cultured in 96 well plates at a density of 2,000 cells/ well. The cells with siRNA transfection or with the inhibitor treatment were monitored for viability using MTT at indicated time points. At the given time points, MTT reagent was added to the media and allowed to incubate for 3 h at 37° C in a humidified incubator at 5% CO2. The medium was then removed and MTT crystals were dissolved in DMSO (Sigma). Cell viability was quantified by reading the plates at an absorbance of 540 nm on a μQuant microplate reader using KC4 data analysis software (Bio-Tek Instruments). The 540 nm absorbance of vehicle-treated wells was used to define 100% proliferation. Each condition was performed in replicates of six wells.

### Determination of inhibition of Aurora and JAK2 kinase activity

Cells were plated in 6 cm dishes at a density of 2 × 10^5^ cells/dish. Aurora A activity was determined by measuring autophosphorylation of Aurora A on Thr288 in cells that were synchronized by treatment with nocodazole (100 ng/mL) for 20 h prior to the treatment with Aurora inhibitors, whereas Aurora B activity was determined by measuring phosphorylation of histone H3 on Ser-10 (p-HH3). DMSO was used as a vehicle control, and Alisertib (0.3 μM) or VX-680 (0.5 μM) was used as a positive control for Aurora inhibition and Ruxolitinib as a positive control for JAK inhibition. Cells were harvested after 2 h of treatment and processed for SDS−PAGE and Western blotting.

### Western blotting

Cells were harvested from experiments of ectopic expression or knockdown using siRNA or the treatment with the inhibitors, washed, and then lysed using lysis buffer as previously described [51]. The protein concentration of the lysate was measured, and the lysate was mixed with gel electrophoresis loading buffer, boiled for 5 minutes, separated by SDS-PAGE, and transferred to nitrocellulose membranes. The membranes were blocked at room temperature for 1 h in TBST containing 5% (w/v) milk before incubating them overnight at 4°C with the following antibodies: anti-Aurora A/AIK (1G4) (Cell Signaling, #4718), Aurora B/AIM1 (Cell Signaling, #3094), p-Aurora A Thr 288 (Cell Signaling, #3079), p-histone H3 (Ser-10) and histone H3 (Cell Signaling, # 9701 and #9715), JAK2 (D2E12) (Cell Signaling, #3230), p-STAT3 (Y705) (Cell Signaling, #9131) and STAT3 (F-2) (Santa Cruz Biotechnology, SC-8019), p-Akt (Ser-473) and Akt2 (D6G4) (Cell Signaling, # 9271 and #3063), p-ERK and ERK (Cell Signaling, #9101 and #9102), as well as cleaved PARP and cleaved caspase-3 (Cell Signaling, #5625 and #9604), and PARP-1 (F2) SC-8007 (Santa cruz Biotechnology) After wash, the membranes were then incubated with secondary antibodies conjugated to horseradish peroxidase (Jackson ImmunoResearch Lab) at room temperature for 1 h and visualized with the ECL system (Perkin-Elmer) as described previously [51].

### Cell-cycle analysis and detection of apoptosis

To evaluate the effect of inhibitors on cell cycle progression, cells were treated with inhibitors at indicated dose or DMSO control and then harvested, fixed with 70% ethanol pretreated with RNase, and stained with propidium iodide (PI) (Sigma) as previously described for flow cytometry analysis [52]. For the detection of apoptosis, cells were cultured for 24 h in medium with inhibitors at indicated dose or DMSO control and harvested for SDS−PAGE and Western blotting. The siRNA or drug treatment-induced apoptosis was detected by Western blot analysis of the cleavage of PARP and caspase-3.

### Soft agar assay

For analysis of anchorage-independent growth, log-phase growing cells were trypsinized, and triplicates of 1.5 × 10^3^ cells per well were seeded in regular growth medium containing 0.3% agar (Sigma) and drug added at the indicated concentrations, as previously described [53]. The cells were allowed to incubate at 37° C for 3 weeks and fed once per week with the inhibitor before 1 mg/ml MTT (Sigma) solution was added overnight to facilitate colony counting. Colonies were scored and counted according to size, as previously described [53].

### Cell Invasion

The invasion capability of the immortalized cells HPNE transfected with either Aurora A or JAK2 or both of them, as well as the tumor cell lines MDA-MB-468, and HT29 treated with siRNA or compounds was measured using Matrigel coated Transwell cell culture chambers (8 μm pore size; Costar, Acton, MA) as described previously [54]. Cells were transfected with Aurora A and JAK2 for 48 h or with siRNA for 72 h or treated with the compounds for 24 h and then were trypsinized and resuspended in serum-free medium and placed in the upper chamber of the Matrigel-coated (0.8 μg/μL, BD Biosciences, San Diego, CA) Transwell insert (from 2 to 5 × 10^4^ cells/well) and with 0.5% DMSO or the compounds at the indicated dose. Medium containing 10%FBS was placed in the lower chamber. Cells were incubated for 48 h in a humidified atmosphere with 95% air and 5% CO2 at 37 °C. Invading cells in the membrane were fixed with 100% methanol and stained with fresh prepared 0.2% crystal violet. Noninvasive cells in the upper chamber were removed by wiping the upper side of the membrane with a cotton swab, and cells located on the underside of the filter were observed and photographed under Olympus stereo microscope. The number of invading cells was counted using Image-Pro analysis software.

### Antitumor study of human tumor xenografts in nude mice

Female nude mice (Charles River Laboratories, Wilmington, MA) were maintained and treated in accordance with the Institutional Animal Care and Use Committee procedures and guidelines. Exponentially growing MDA-MB-468 cells were harvested via trypsinization, pelleted at 400 g for 5 min. Cell pellets were re-suspended in DPBS (Invitrogen) at 10 x10^6^ cells per 200 μl, and injected into right flank of mice. The tumor xenografts were monitored with an electronic caliper measurements and tumor volume (*V*) was calculated using the formula V= (W × L^2^)/2, where width is the largest diameter and length is the smallest diameter. When the tumors reached ~ 250 mm^3^, the animals were randomized and treatment schedules were implemented. Treatments consisted of intraperitoneal (i.p.) injections of vehicle control (50% polyethylene glycol (PG) + 15% 2-hydroxypropyl-beta-cyclodextrin (HPCD) (n=5) or AJI-100 (n=6) at 50 mg/kg, everyday for 14 days.

### Statistical analysis

Graphs were prepared using GraphPad prism software. In this study, Student's *t*-tests were used to determine the statistical significance of the data of soft agar assay and cell invasion assay between transfected (ectopic genes and specific siRNA) or treated (compounds) cells and control cells (vector, NT siRNA and vehicle). A *P*-value < 0.05 was chosen as a threshold for statistical significance.

## SUPPLEMENTARY FIGURES


