# Supplementary material for: Dual Aurora A and JAK2 kinase blockade effectively suppresses malignant transformation
Source: Oncotarget. 2014 Mar 22;5(10):2947–61. doi: 10.18632/oncotarget.1615 (PMC4102782; doi:10.18632/oncotarget.1615)
Supplement: Supplementary file 1 [file oncotarget-05-2947-s001.pdf]

SUPPLEMENTARY FIGURES

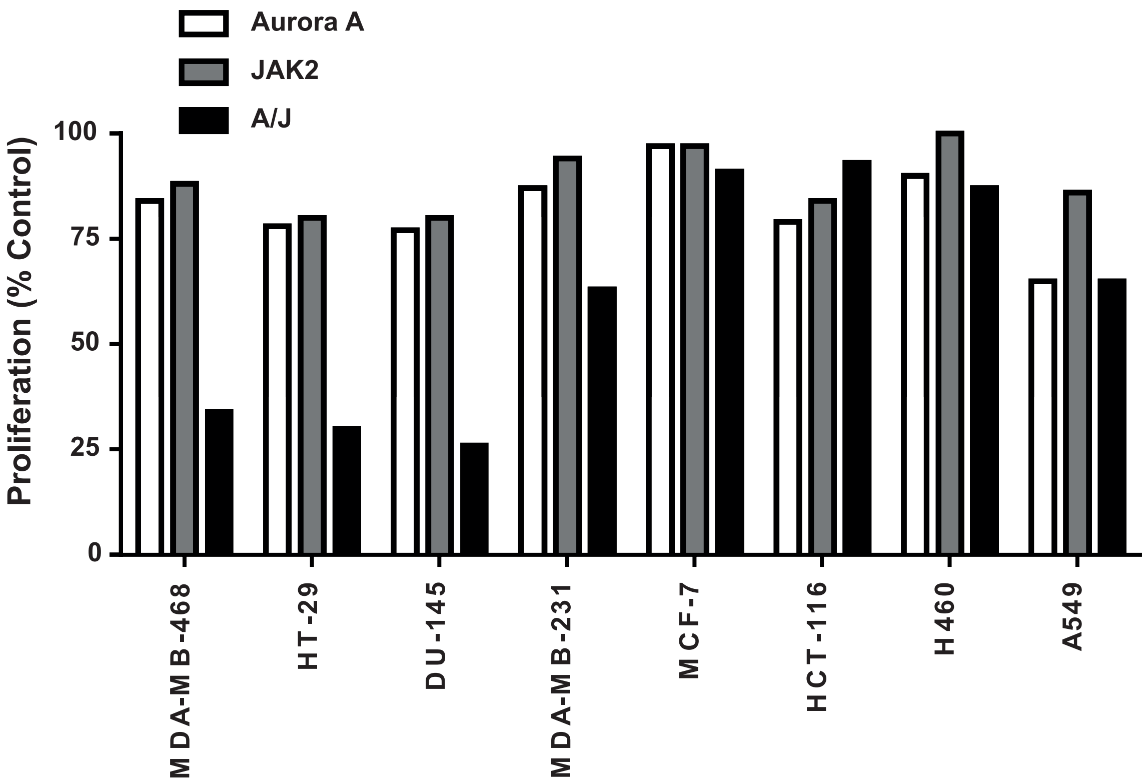

Figure S1: Effects of single vs. combination knockdown of Aurora A and JAK2 on proliferation of 8 human cancer cell lines. The cell lines were transfected with 35 nM of siRNA for NT, Aurora-A, JAK2 or both. Cells were harvested 72 h post-transfection, counted and seeded at 1,000 cells per well in 96-well plates. Anchorage-dependent cell proliferation was assessed by MTT assay after 96 h as described under Materials and Methods. The data are representative of 2 independent experiments.

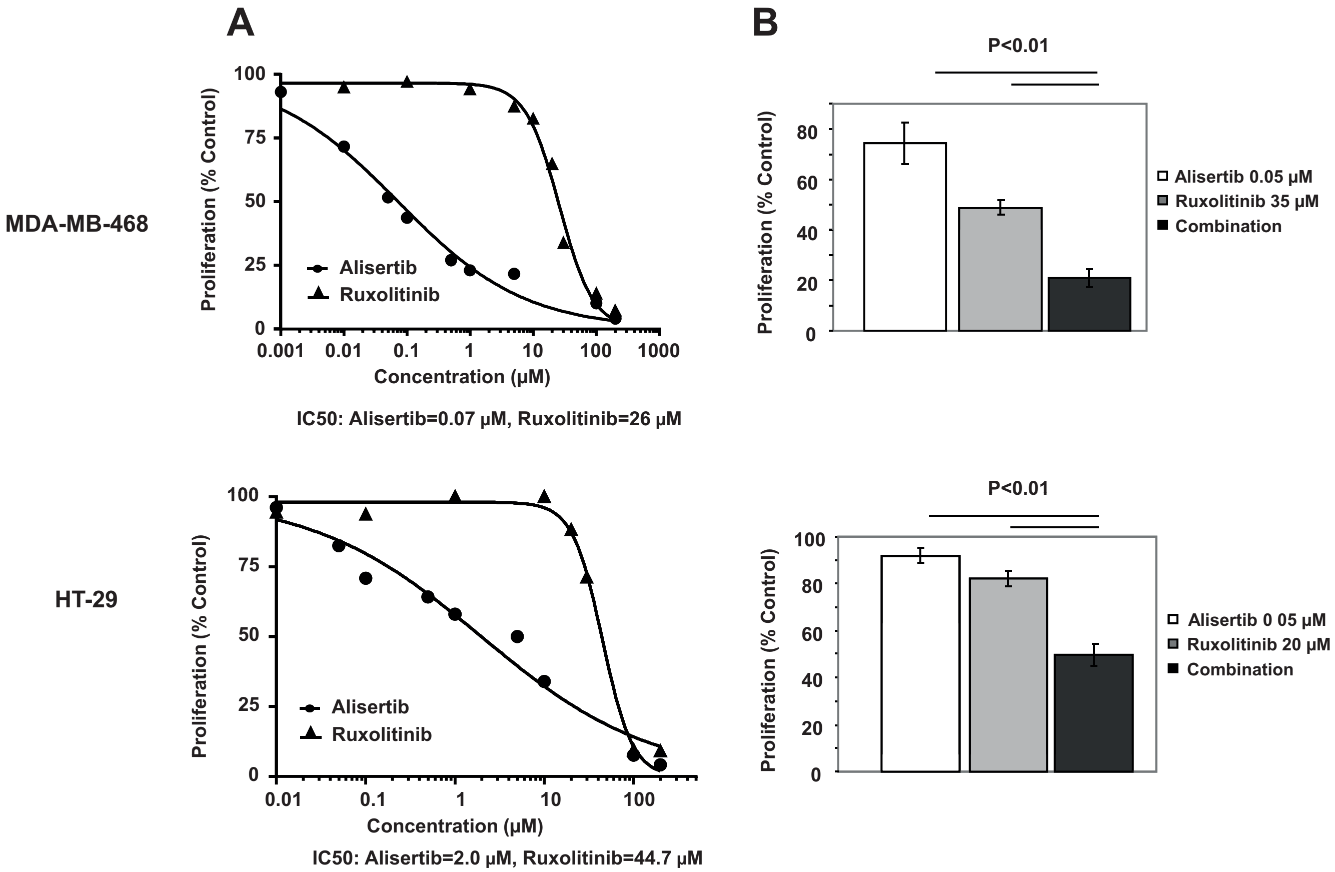

Figure S2: Combination treatment of Alisertib and Ruxolitinib is more effective than single treatment at inhibiting cell proliferation in MDA-MB-468 and HT-29 cells. (A) Cells were plated in 96-well plates and treated with the indicated concentrations of Alicertib and Ruxolitinib for 48 h and processed for MTT assays as described under Materials and Methods. **(B)** Cells were treated with Alisertib and Ruxolitinib either as single agents or in combination at the indicated dose for 48 h and processed for MTT assays. The data for (A) and (B) are representative of three independent experiments.

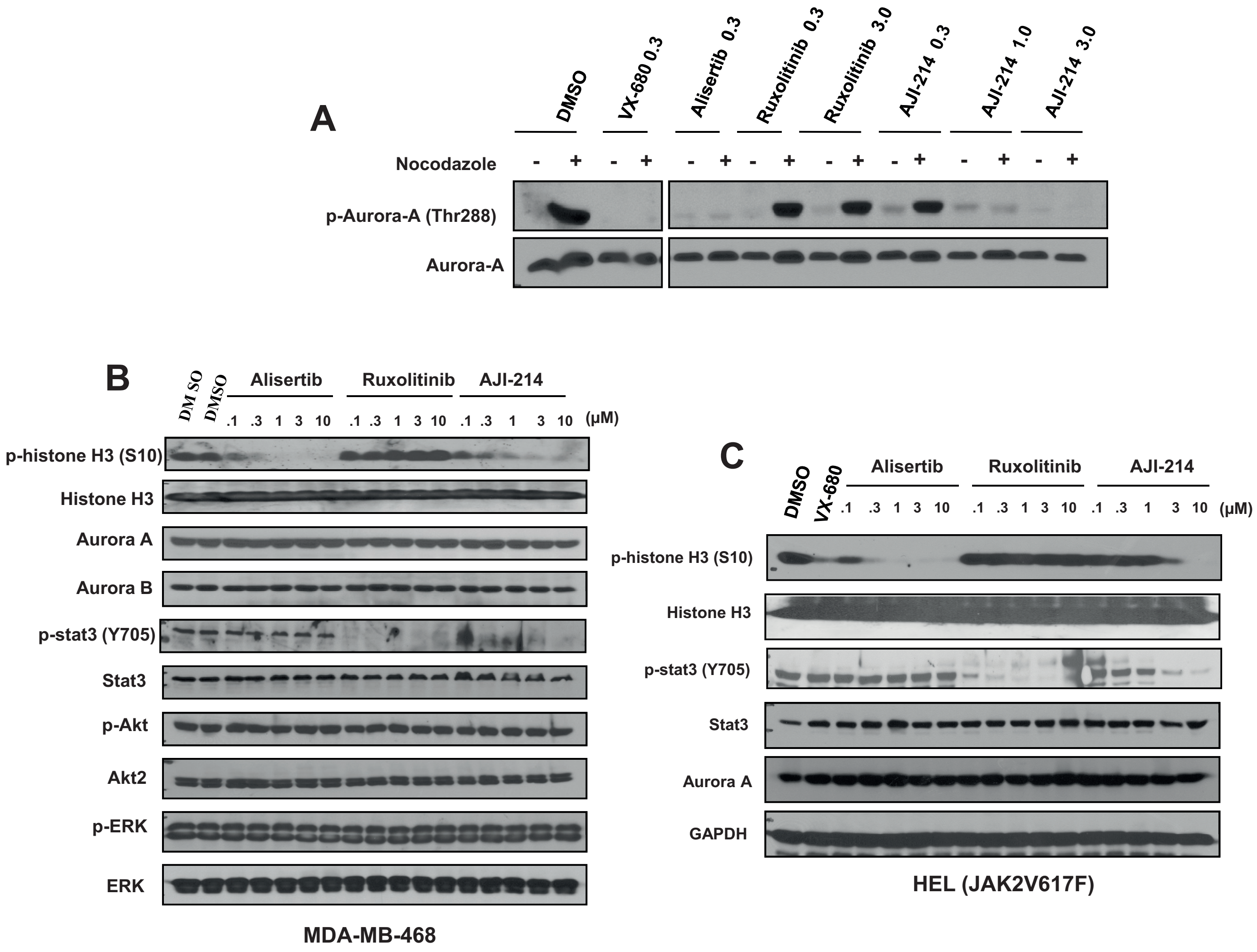

Figure S3: Effects of Alisertib, Ruxolitinib and AJI-214 on the phosphorylation of Aurora A, Histone H3, STAT3, Akt and Erk in breast cancer and leukemia cells. (A) MDA-MB-468 cells were synchronized by treatment with nocodazole and then treated with the compounds at the indicated concentration for 2 h as described under Materials and Methods. Cells were then harvested and processed for Western immunoblotting with p-Aurora-A (Thr288) and total Aurora A antibodies. MDA-MB-468 **(B)** and Leukemia HEL (JAK2V617F) **(C)** cells were treated with Alicertib, Ruxolitinib and AJI-214 at the indicated concentration for 2 h, harvested and processed for Western immunoblotting with the indicated antibodies as described under Materials and Methods. Data for (A), (B), (C) are representative of two independent experiments.

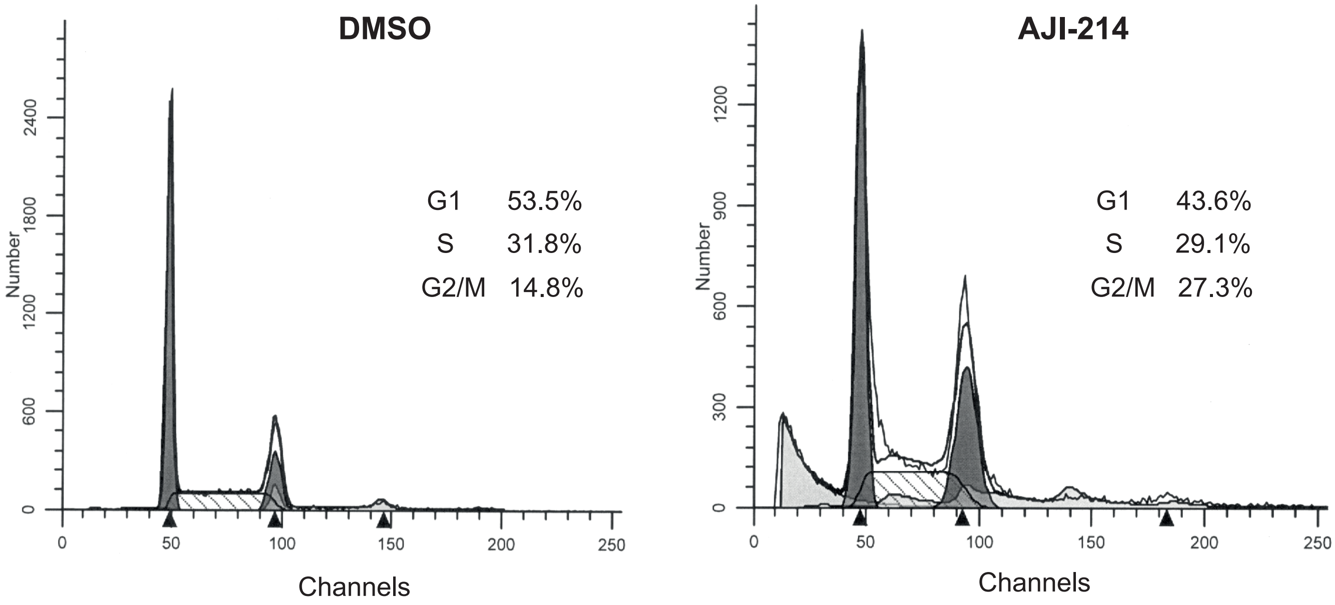

Figure S4: AJI-214 treatment induces the accumulation of MDA-MB-468 cells in the G2/M phase of the cell cycle. MDA-MB-468 Cells were treated with vehicle or AJI-214 for 24h, and then harvested for cell-cycle analysis as described under Materials and Methods. Data are representative of two independent experiments.
